# Transformers deep learning models for missing data imputation: an application of the ReMasker model on a psychometric scale

**DOI:** 10.3389/fpsyg.2024.1449272

**Published:** 2024-12-17

**Authors:** Monica Casella, Nicola Milano, Pasquale Dolce, Davide Marocco

**Affiliations:** ^1^Natural and Artificial Cognition Laboratory, Department of Humanistic Studies, University of Naples “Federico II”, Naples, Italy; ^2^Department of Translational Medical Science, University of Naples “Federico II”, Naples, Italy

**Keywords:** missing data, machine learning, artificial intelligence, deep learning, psychometrics

## Abstract

**Introduction:**

Missing data in psychometric research presents a substantial challenge, impacting the reliability and validity of study outcomes. Various factors contribute to this issue, including participant non-response, dropout, or technical errors during data collection. Traditional methods like mean imputation or regression, commonly used to handle missing data, rely upon assumptions that may not hold on psychological data and can lead to distorted results.

**Methods:**

This study aims to evaluate the effectiveness of transformer-based deep learning for missing data imputation, comparing ReMasker, a masking autoencoding transformer model, with conventional imputation techniques (mean and median imputation, Expectation–Maximization algorithm) and machine learning approaches (K-nearest neighbors, MissForest, and an Artificial Neural Network). A psychometric dataset from the COVID distress repository was used, with imputation performance assessed through the Root Mean Squared Error (RMSE) between the original and imputed data matrices.

**Results:**

Results indicate that machine learning techniques, particularly ReMasker, achieve superior performance in terms of reconstruction error compared to conventional imputation techniques across all tested scenarios.

**Discussion:**

This finding underscores the potential of transformer-based models to provide robust imputation in psychometric research, enhancing data integrity and generalizability.

## 1 Introduction

Dealing with missing data represents a significant challenge in psychological research and other scientific fields. Missing data can arise for various reasons, including human error, data processing issues, participant non-response, or unobserved variables.

The presence of missing data complicates data analysis significantly, both in an explanatory context, where the goal is to estimate unbiased model parameters and draw inferences, and in a predictive context, where the aim is to develop algorithms capable of recognizing hidden patterns and providing accurate predictions for output values based on new input data (Breiman, 2001; Shmueli, 2011; Yarkoni and Westfall, 2017).

In the context of psychological research, missing data introduces unique challenges that affect both explanatory and predictive analyses. From an explanatory point of view, the challenges introduced by missing data are twofold: they reduce statistical power and lead to biased parameter estimates (Roth, 1994). In particular, reduced statistical power weakens the sensitivity of statistical tests to detect relationships within the data, typically requiring larger sample sizes to compensate for the loss (Schmidt et al., 1976). Even minor data loss can significantly impact power: for instance, a small percentage of randomly missing data handled via listwise deletion can reduce effective sample sizes by a substantial margin (Kim and Curry, 1977), compounding the power limitations inherent in psychological studies that often face practical constraints on sample size (Kenny et al., 2002).

Moreover, missing data poses a substantial risk to the accuracy of parameter estimates, potentially leading to biased conclusions that misrepresent underlying relationships in the data. This bias is especially problematic in applied psychology, where real-world factors can lead to selective data loss. For example, missing data from high or low ends of a distribution can distort measures of central tendency and variability, skewing results that rely on accurate estimates of population parameters (Little and Rubin, 2019). Such issues are not trivial: biased estimates can misinform theoretical interpretations and compromise the practical utility of models used in applied settings.

The presence of missing data is challenging also from a predictive perspective. Indeed, incorrect imputation of an influential predictor can significantly reduce prediction performance. Moreover, improper imputation can distort the relationships among inputs, introducing noise and deteriorating the performance of the prediction algorithm. Therefore, it is crucial to have a validated and robust approach for handling such instances (Fletcher Mercaldo and Blume, 2020).

A cornerstone concept in missing data literature is the classification of missing data techniques, which relates the likelihood of data being missing to the characteristics of subjects or variables (Nakagawa, 2015). The most famous framework for missing mechanisms (Rubin, 1976; Little and Rubin, 2019), delineates three types of missing data: Missing Completely at Random (MCAR), Missing at Random (MAR), and Missing Not at Random (MNAR). While MCAR assumes no relationship between missingness and observed or unobserved variables, making it a simplistic starting point for imputation, it is rarely applicable in practical settings. In contrast, MAR, which considers missingness dependent on observed variables, is a more realistic assumption for most psychological studies. When neither MCAR nor MAR assumptions hold, missing data is classified as MNAR, where missingness depends on unobserved values.

Historically, a wide array of strategies has been developed in several research fields to address the issue of missing data, with techniques ranging from explanatory statistical methods to predictive machine learning algorithms, and more recently, sophisticated deep learning approaches (Sun et al., 2023).

Conventional methods used in psychometric research are based prevalently on an explanatory perspective (e.g., mean/median imputation, expectation–maximization). However, these techniques often rely on assumptions that may not hold true in psychological contexts, such as the linearity of relationships or the normality of data distributions. In particular, as discussed by Sun et al. (2023), although conventional imputation techniques such as mean/median imputation are computationally efficient and easy to implement, they present several disadvantages. Mean/median imputation often underestimates variability, leading to overly simplistic imputations, while regression imputation may struggle to capture complex or nonlinear relationships within the data. While Expectation–Maximization (EM) provides a more refined solution, it can be computationally demanding and prone to slow convergence, particularly when applied to large datasets.

On the other hand, predictive techniques, such as machine learning and deep learning methods, have increasingly been recognized as valuable for missing data imputation across various scientific fields (e.g., Pantanowitz and Marwala, 2009; Hallaji et al., 2021; Qiu et al., 2020). Machine learning approaches offer a more dynamic and adaptable framework for managing missing data since they require mild assumptions.

Random forests and K-nearest neighbors (KNN) algorithms have been particularly notable among the machine learning approaches. Random forests address missing data by using an ensemble of decision trees which work together to estimate missing values based on the similarities within the data (Stekhoven and Bühlmann, 2012). This method is robust against overfitting and can handle large datasets with complex structures. KNN, on the other hand, estimates missing values based on the proximity to the nearest neighbors in the dataset, assuming that similar data points (or neighbors) are likely to have similar values (Batista and Monard, 2002).

Among the most recently advanced machine learning techniques, deep learning models, which are multi-layered artificial neural networks, have emerged as promising tools for missing data imputation. In general, and as discussed by the review of Sun et al. (2023), deep learning models are powerful tools which can capture complex, nonlinear relationships without needing predefined rules or strict distributional assumptions. This flexibility allows deep learning models to work effectively with high-dimensional datasets and diverse types of data. Moreover, their ability to learn from latent structures in the data gives them a significant advantage in capturing hidden patterns that conventional methods might miss.

Recent research in deep learning has introduced sophisticated neural network architectures equipped with attention mechanisms. One notable example is the ReMasker architecture, introduced by Du et al. (2023). The ReMasker method addresses naturally missing values in datasets and randomly selects and “re-masks” additional values. The autoencoder is optimized to reconstruct this re-masked set, and the trained autoencoder is then used to predict the missing values.

Compared to prior methods, ReMasker offers several advantages. It utilizes the Transformer architecture (Vaswani et al., 2017) as its backbone, employing the self-attention mechanism to capture intricate inter-feature correlations (Huang et al., 2020). While the Transformer model is the standard architecture for building large language models and has led to the development of pre-trained systems such as generative pre-trained transformers (GPTs) (Radford et al., 2018, 2019; Brown et al., 2020; OpenAI et al., 2023) and BERT (Bidirectional Encoder Representations from Transformers) (Devlin et al., 2018), it also has applications in computer vision, audio, and multi-modal processing (e.g., Wu et al., 2020; Radford et al., 2022; Xu et al., 2023).

In particular, the self-attention mechanisms enable the network to dynamically weigh the importance of different variables, which is crucial when dealing with incomplete datasets. Unlike traditional methods, these models do not rely on predefined assumptions about the nature of missingness, making them highly adaptable and effective across various types of data and missing data scenarios. Consequently, these approaches are not only more accurate but also more flexible, adapting to the specific characteristics and requirements of the dataset at hand.

Despite the increasing presence and recognition of machine learning and deep learning techniques in psychological research as valuable alternatives to traditional psychometric methods (e.g., Gonzalez, 2021; Urban and Bauer, 2021; Casella et al., 2024; Dolce et al., 2020; Luongo et al., 2024), including in addressing missing data imputation challenges (e.g., Collier et al., 2024; Yoon et al., 2018), comprehensive comparisons remain scarce, particularly in psychometric contexts where the use of predictive algorithms for missing data imputation is still limited.

In this study, we evaluate the REMASKER transformer architecture, introduced by Du et al. (2023), by comparing it with traditional imputation methods (mean/median imputation), machine learning approaches (K-nearest neighbors and MissForest) and an Artificial Neural Network model named autoencoder. Our comparisons are conducted on simulated missing data within a real psychological dataset under the Missing Completely at Random (MCAR) and Missing at Random (MAR) assumptions, using numerical experiments that vary sample sizes and missing data ratios. For this work, we do not consider Missing Not at Random (MNAR) assumptions, as they require specific considerations and methods that are beyond the scope of this study.

## 2 Methods

In this section, we provide a concise overview of the imputation methods being examined and introduce the REMASKER model. We then detail the dataset utilized in our study and describe the process employed to simulate missing data.

### 2.1 Statistical methods for missing imputation

One of the most commonly used imputation techniques is mean and median imputation. These foundational methods are employed to handle missing data in statistical analysis. They operate under the assumption that the missing values can be approximated using the central tendency of the observed data. Specifically, for continuous variables, the mean (arithmetic average) or median (middle value in the dataset) of observed values is calculated and used to fill in missing entries. For categorical variables, the mode (most frequently occurring value) is used. These methods are straightforward but can introduce bias, particularly if the missing data are not Missing Completely at Random (MCAR). This bias occurs because these imputation methods do not account for the variability in the data, potentially leading to underestimated variances and covariances in the imputed dataset (Schafer and Graham, 2002).

Another statistical method for missing data imputation is based on the Expectation–Maximization algorithm, which was proposed by Dempster et al. (1977) to handle missing data based on the maximum likelihood estimation of parameters. The EM method is an iterative process (Little and Rubin, 2002). Each iteration of EM consists of an expectation (E) step and a maximization (M) step. In the E step, the conditional expectation of the complete data log-likelihood is derived in the presence of the observed data and the current estimates for parameters. In the M step, the conditional expectation of the complete data log-likelihood is maximized in order to yield a new set of parameter estimates. The E and M steps iterate until the difference in the observed log-likelihood from two consecutive iterations meets a prespecified convergence criterion. When the EM algorithm converges, a final set of estimates for parameters (e.g., means, variances/covariances) are obtained. From these estimates, expected values for the missing data can be derived from the EM algorithm. EM is easy to implement and stable (Couvreur, 1997), and has been shown to yield unbiased estimates of parameters when its assumptions (i.e., multivariate normality and MAR) are met (Barnard et al., 2000; Schafer, 1997). The flexibility of EM in model specification and its ability to handle the uncertainty of parameter estimates in incomplete data scenarios make it a powerful tool for complex datasets (McLachlan and Peel, 2000).

### 2.2 Machine learning methods for missing imputation

Over the years, many Machine Learning methods have been proposed for missing data imputation. Among them, K-Nearest Neighbors and Random Forests are the most used and widely known in several scientific fields.

In particular, the K-Nearest Neighbors (KNN) algorithm is utilized for imputation by identifying the k-nearest neighbors for each data point with a missing value, using a specific distance metric, often the Euclidean distance (Batista and Monard, 2002). This method involves computing the proximity between data points and selecting the nearest neighbors based on the chosen metric. The missing values are then imputed using either the mean (for continuous variables) or mode (for categorical variables) of the neighbors’ values.

The success of KNN imputation largely depends on the choice of two crucial parameters: the number of neighbors (*k*) and the distance metric used. The selection of *k* is critical; a small *k* may lead to high sensitivity to noise, potentially causing the imputation to reflect outliers rather than the true distribution of data. On the other hand, a large *k* may decrease the algorithm’s sensitivity to specific data patterns and increase computational demands, as more neighbors are considered in the imputation process. One significant drawback of the KNN algorithm is its computational expense. The algorithm requires calculating the distance between each pair of points in the dataset, which can become computationally intensive as the size of the dataset increases. This process involves repeatedly measuring distances and sorting or ranking these distances to determine the closest neighbors, which can be particularly demanding in terms of both time and computational resources. Moreover, the effectiveness of the KNN algorithm is also affected by the structure of the data. The algorithm is particularly suitable for datasets with a limited number of variables that exhibit strong correlations. Under these conditions, KNN can more accurately identify the genuine similarities between observations, reflecting these in the imputed values.

The MissForest imputation methods (Stekhoven and Bühlmann, 2012) utilize an ensemble of decision trees (specifically, random forests) to predict missing values, iteratively imputing each feature based on the others. This non-parametric approach handles mixed-type data effectively, adapting to the intrinsic structure of the data. Unlike simpler imputation methods, MissForest incorporates randomness and ensemble learning to capture complex interactions and non-linearities in the data. Each iteration of MissForest refines the imputation, using out-of-bag (OOB) error as a convergence criterion. This method has been shown to be robust across various scenarios, including those with high dimensions and substantial interaction between features (Stekhoven and Bühlmann, 2012).

The missForest algorithm starts by initializing missing values in a variable, replacing them with the mean for continuous variables or the most frequent class for categorical variables. The imputation process then proceeds sequentially through the dataset, ordered by the number of missing observations in each variable. The variable being imputed serves as the response for constructing the random forest model. The dataset is split into two groups: one with observed values used as the training set, and another with missing values used as the prediction set. Then, the random forest models predict and replace the missing values for the variable under imputation. After all variables with missing data have been imputed, one iteration is complete. This iterative process continues until the relative sum of squared differences (or the proportion of falsely classified entries for categorical variables) between the current and previous imputation results increases. At this point, the missForest algorithm outputs the prior imputation as the final result.

### 2.3 Artificial neural networks for missing data imputation: the autoencoder and the ReMasker model

Among the artificial neural networks proposed over the years to handle missing data imputation, this contribution focuses on the autoencoder and the ReMasker model, two prominent approaches that rely on neural network architectures to address the challenges of missing data.

**Figure 1:**
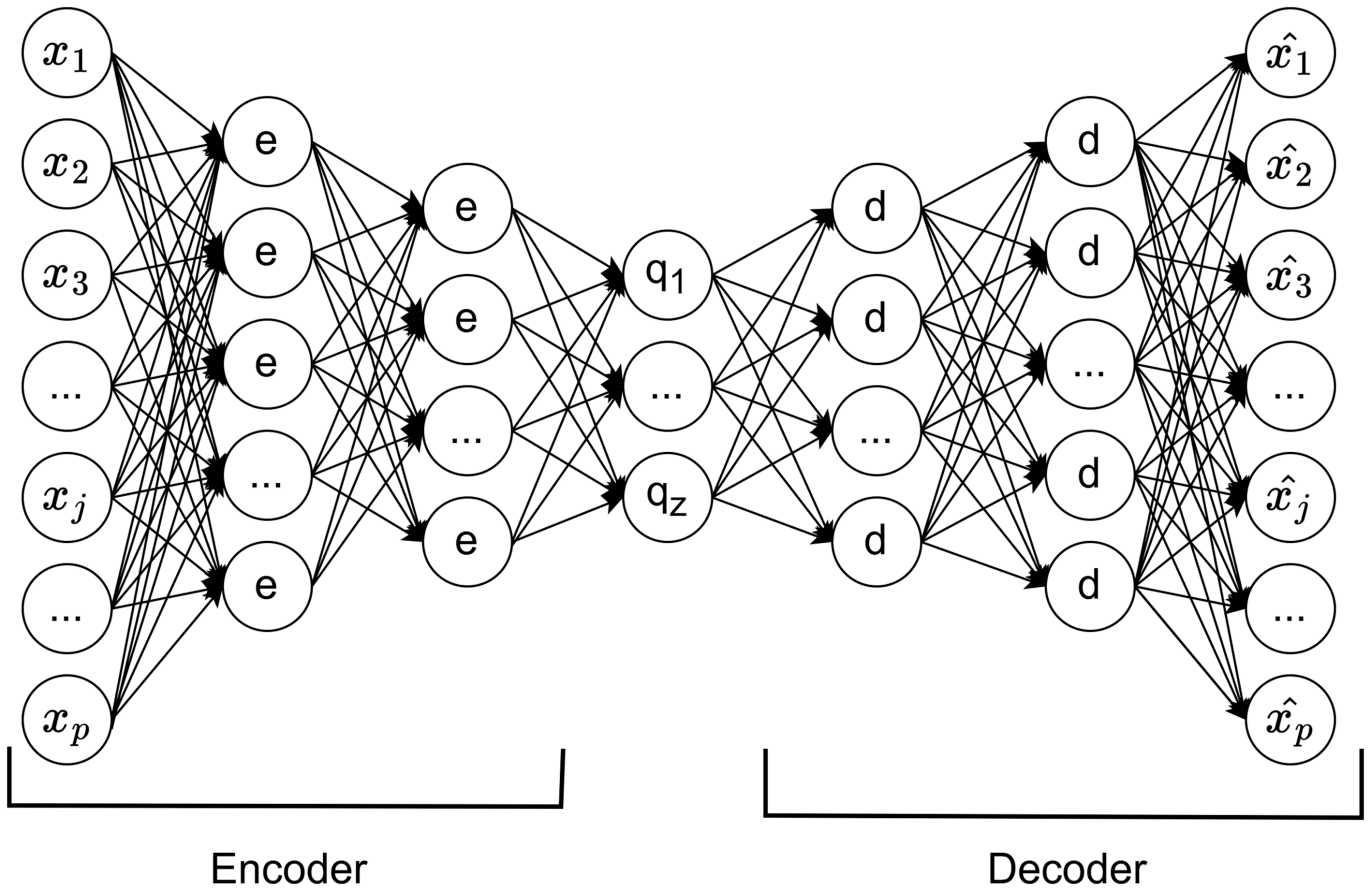
Example of an autoencoder model structure.

An autoencoder is a multi-layer perceptron with the same number of input and output units but fewer hidden units (Bourlard and Kamp, 1988). During training, the output units aim to match the input values, allowing the network to learn representations in a self-supervised manner. The encoder maps an input to a representation, and the decoder reconstructs the original input, with the central hidden layer encoding the most relevant information for this reconstruction (see Figure 1).

Introducing nonlinear hidden layers allows the network to perform nonlinear dimensionality reduction (Kramer, 1991). Autoencoders have diverse applications, including facial recognition and customer segmentation (Siwek and Osowski, 2017; Alkhayrat et al., 2020). In the context of missing data imputation, an autoencoder learns the underlying data representation and relationships, allowing it to reconstruct missing values effectively. In particular, the autoencoder reconstructs missing data points based on their relationships with other observed features, making it particularly useful in scenarios where data exhibit nonlinearity and high-dimensional complexity.

Building upon the foundation of autoencoders, the ReMasker model extends this approach and incorporates more sophisticated techniques for missing data imputation (Du et al., 2023). The ReMasker model is based on a masked autoencoding framework and a Transformer architecture.

The core innovation of ReMasker is its re-masking strategy, which involves masking not only the naturally missing values but also a randomly selected set of observed values. The model learns to reconstruct these re-masked values during training, enabling it to predict the originally missing values with high fidelity.

ReMasker builds on the concept of masked autoencoders (MAE), initially developed for natural language processing (NLP) and later applied to computer vision (He et al., 2022). In NLP, Masked modeling in NLP involves masking a subset of input tokens and training the model to predict these masked tokens using the surrounding context. This approach enhances the model’s understanding of language patterns and dependencies. Similarly, the MAE partially masks input data during training, compelling the model to reconstruct the missing parts. This method helps the model learn more robust and generalized features, enhancing its performance in downstream tasks. Figure 1 shows an example of masked modeling in the context of natural language processing.

**Figure 2:**
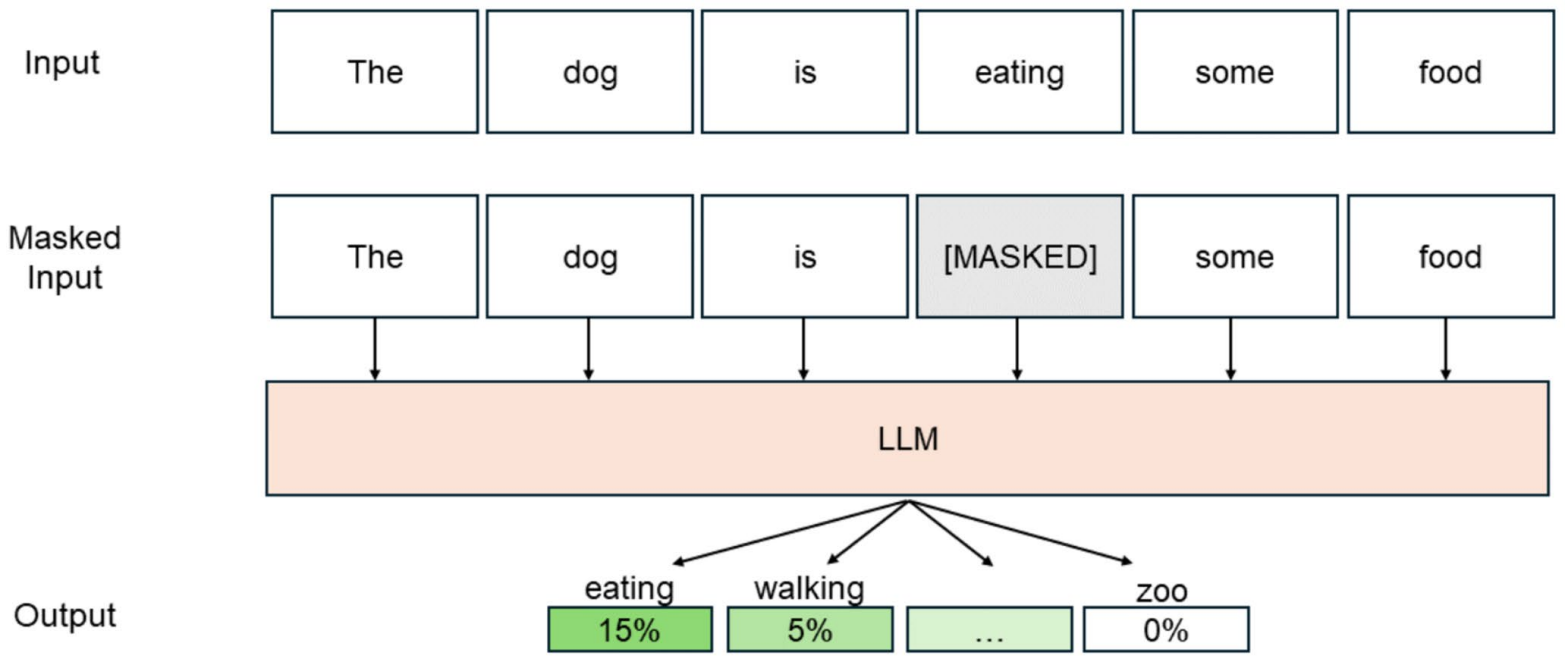
An example of a masked modeling framework for natural language processing.

Starting from this framework, the ReMasker model also uses a Transformer architecture, using the Transformer’s self-attention mechanism to capture complex inter-feature correlations (Vaswani et al., 2017). This design allows the model to handle various missing data scenarios effectively without specific assumptions about the missingness mechanisms (Figure 2).

An attentional mechanism in a neural network selectively focuses on parts of its input data that are more relevant for a specific task, similar to how humans pay attention to certain aspects of a visual scene or conversation. In neural networks dealing with sequences or complex data relationships (such as NLP or time-series analysis), the attention mechanism dynamically weighs the importance of different input features, enabling the model to prioritize data processing.

The attention mechanism works by creating a set of scores (often through a small neural network) that determine the focus on each input component. These scores produce a weighted sum of the input features, where more important features get a higher weight, representing what the model pays the most attention to for further processing or making predictions.

In data imputation, using an attentional mechanism allows the model, such as ReMasker, to better understand and represent underlying data patterns. It can discern which features most indicate the nature of a missing value and use this information to predict missing values accurately. This is particularly beneficial in complex datasets where feature relationships are not straightforward and can vary significantly.

The ReMasker model’s Transformer architecture employs an advanced form of attention mechanism known as multi-head self-attention, allowing the model to handle data with varying patterns of missingness effectively. Each “head” of attention can focus on different data relationships, providing a comprehensive understanding that aids in accurately imputing missing values.

**Figure 3:**
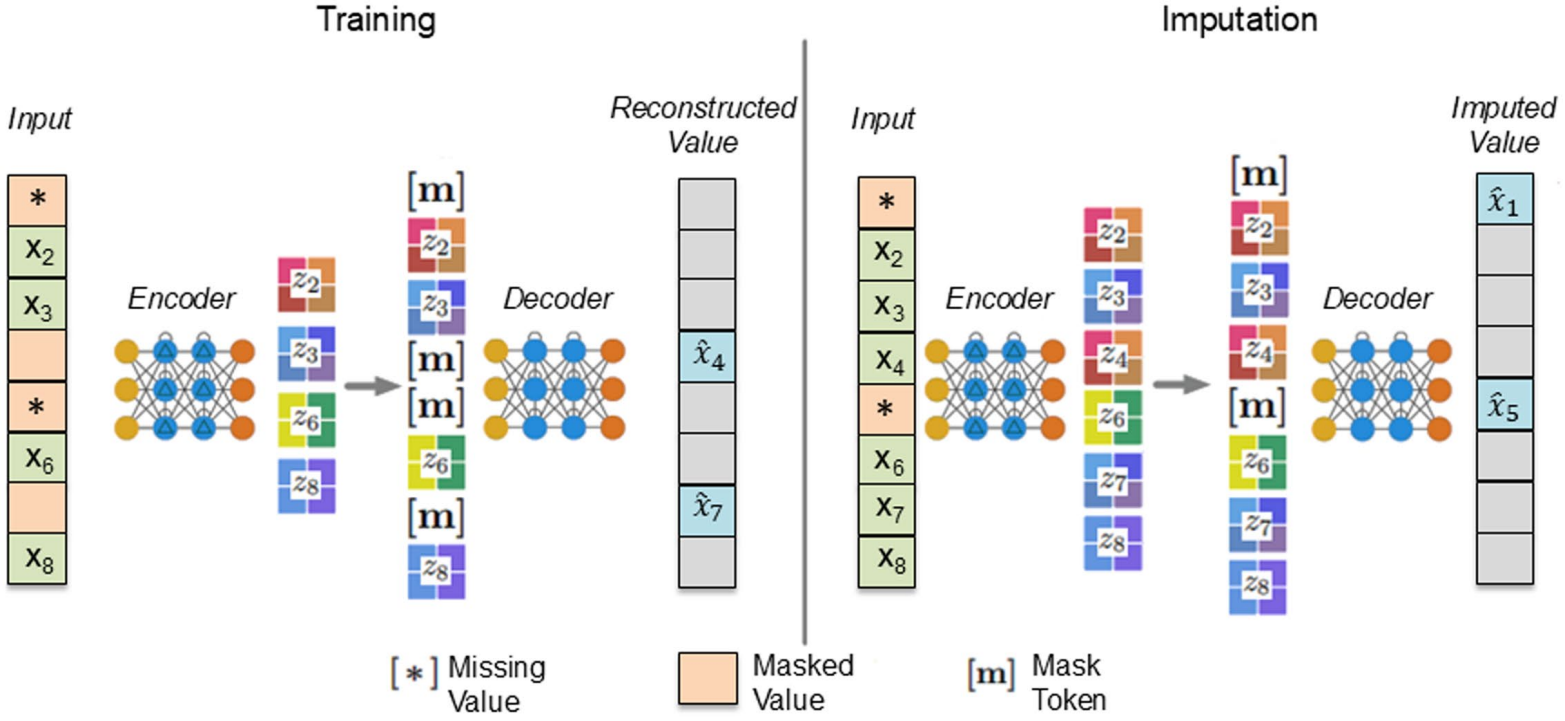
Fitting and imputation of the ReMasker model from Du et al. (2023).

During the training stage, for each input, some values are randomly selected and masked out, in addition to any existing missing values. The encoder processes the remaining values to create an embedding. This embedding is padded with mask tokens and then used by the decoder to reconstruct the masked values. Padding involves adding special elements (padding tokens) to sequences to make them the same length, allowing efficient processing. Tokens are basic units of data, and mask tokens hide certain values in a sequence, which the model learns to predict. In the implementation stage, the optimized model predicts the missing values. Figure 2 from Du et al. (2023) summarizes the training process and the missing value imputation of the ReMasker model. For further details on the ReMasker’s functioning, refer to the work of Du et al. (2023) (Figure 3).

### 2.4 Dataset and missing generation

In our research, we employed the COVIDiSTRESS diverse dataset (Blackburn and Vestergren, 2022), specifically focusing on the Perceived Stress Scale-10 (Cohen and Williamson, 1988) to measure psychological stress.

This dataset, available through the Open Science Framework, includes responses from 20,601 participants from 137 countries, with a final cleaned dataset of 15,740 individuals who met the inclusion criteria, such as being 18 years or older and passing attention checks.

The PSS-10 is a widely recognized scale that evaluates how much individuals perceive situations in their lives as stressful. It consists of 10 items rated on a 5-point Likert scale.

We generate three different sample sizes for this study, drawing from the final cleaned dataset 200, 500 and 1,000 observations.

For the generation of missing data mechanisms (MCAR and MAR), we referred to the study of Muzellec et al. (2020).

In particular, for the MCAR scenario, each value is masked based on the outcome of a Bernoulli random variable with a set parameter. In the MAR scenario, for each experiment, we select a subset of variables that will not have missing values. The remaining variables have missing values generated according to a logistic model with random weights, taking the non-missing variables as inputs. A bias term is adjusted through a line search to achieve the desired percentage of missing values.

The literature does not specify a definitive cutoff for an acceptable percentage of missing data for valid statistical inferences. For instance, Schafer (1999) suggested that a missing rate of 5% or less is negligible, while Bennett (2001) stated that statistical analysis could be biased if more than 10% of data are missing. Moreover, the missing data problem is not solely judged by the amount of missing data. Tabachnick et al. (2013) argued that the mechanisms and patterns of missing data have a more substantial impact on research outcomes than the proportion of missing data. Our experiments included missing rates of 5, 10, and 15%.

For each dataset, all methods were assessed using 10 different sets of missing value masks.

All methods compared were used for continuous data imputation.

The imputation methods were assessed using the root mean square error (RMSE) metric. Specifically, we calculated the RMSE between the original complete matrix and the imputed matrix generated by each method. Before calculating the RMSE, the imputed values were rounded to the nearest integer to align them with the original ordinal scale, ranging from 1 to 5. The RMSE was calculated as follows:


$$\mathrm{RMSE}\;\left(x_{ij},{\hat{x}}_{ij}\right)=\sqrt{\frac{1}{n}\overset{n}{\underset{i=1}{\sum }}\left(\frac{\sum_{j=0}^{p}{\left(x_{ij}-{\hat{x}}_{ij}\right)}^{2}}{p}\right)},\begin{array}{c} i=1,2,\ldots ,n;\\ j=1,2,\ldots ,p \end{array}$$

(1)where $x_{ij}$ is a generic observation of the original matrix for subject i and variable j , and ${\hat{x}}_{ij}$ is the imputed value of the same observation in the imputed matrix. Here, n represents the number of subjects, and p represents the number of inputs.

## 3 Algorithms implementation and results

The algorithms were implemented using R, with the exceptions of KNN, the autoencoder, and REMASKER, which were implemented in Python.

Mean and Median imputation of missing data was performed replacing missing values with the corresponding column mean or median.

The Expectation Maximization (EM) imputation algorithm is implemented using the R package missMethods. Initially, parameters are estimated and then employed in regression-like models to impute missing values. Residuals, drawn from a multivariate normal distribution, are added to the expected values. The algorithm is set to a maximum of 1,000 iterations or until the stopping criterion, defined as the maximum relative difference in parameter estimates falling below 0.0001, is met.

The MissForest algorithm is implemented using the MissForest package in R (Stekhoven, 2011). It runs for a maximum of 10 iterations unless the stopping criterion is satisfied earlier, and constructs 100 trees in each forest.

The KNN imputation algorithm is implemented in Python using the scikit-learn library (Pedregosa et al., 2011). It is configured with three neighbors and calculates distances using the Euclidean metric. A uniform weight function is applied during prediction.

The autoencoder model was implemented in Python using the PyTorch library. The autoencoder architecture consisted of an encoder-decoder structure. Specifically, the encoder included two fully connected layers: the first reduced the input dimension to 7 hidden units with a ReLU activation, and the second layer further reduced it to 5 units, with a ReLU activation. The central layer included 3 nodes with a linear activation function. The decoder mirrored this structure, reconstructing the data back to its original dimension. The model was trained to minimize reconstruction error using the mean squared error (MSE) loss function, with the Adam optimizer and a learning rate of 0.001 for 100 epochs. The autoencoder was trained on masked datasets, where missing values were replaced with zeros during training.

The ReMasker model is implemented in Python and utilizes the architecture and parameters described in the work of Du et al. (2023).

**Figure 4:**
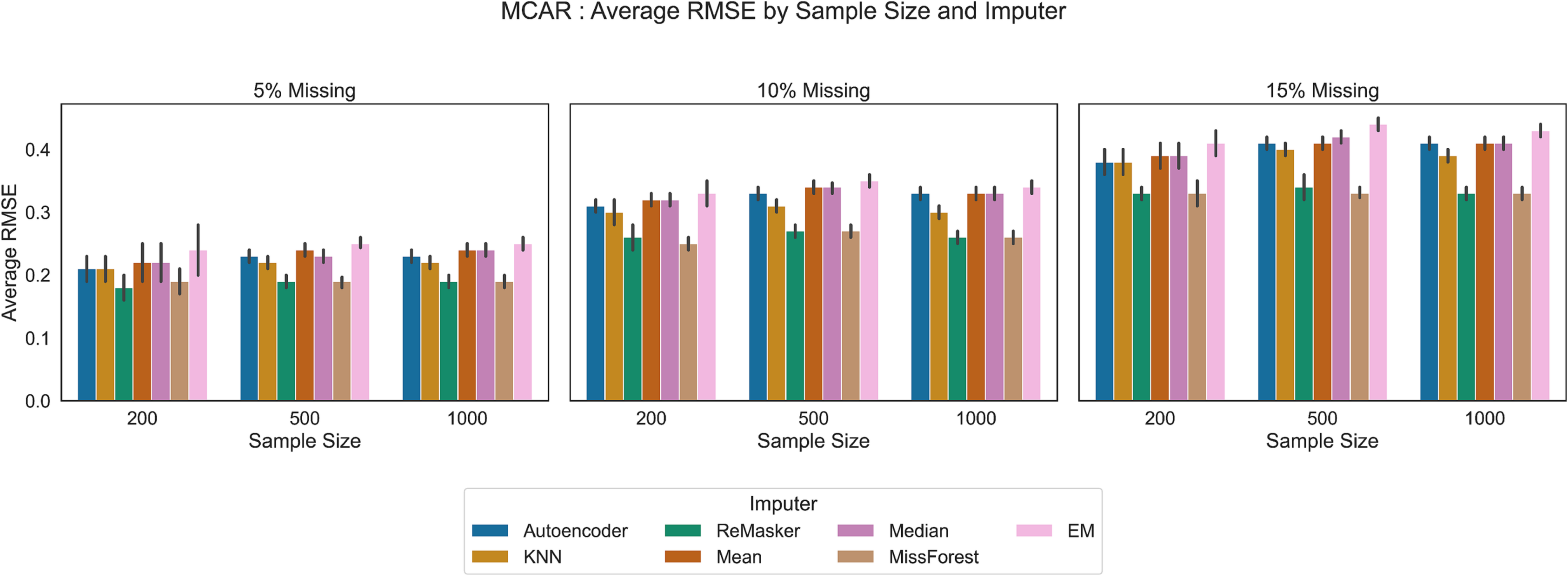
RMSE values in the MCAR scenario for all the tested imputation techniques, under varying conditions of sample size and missing ratio.

**Figure 5:**
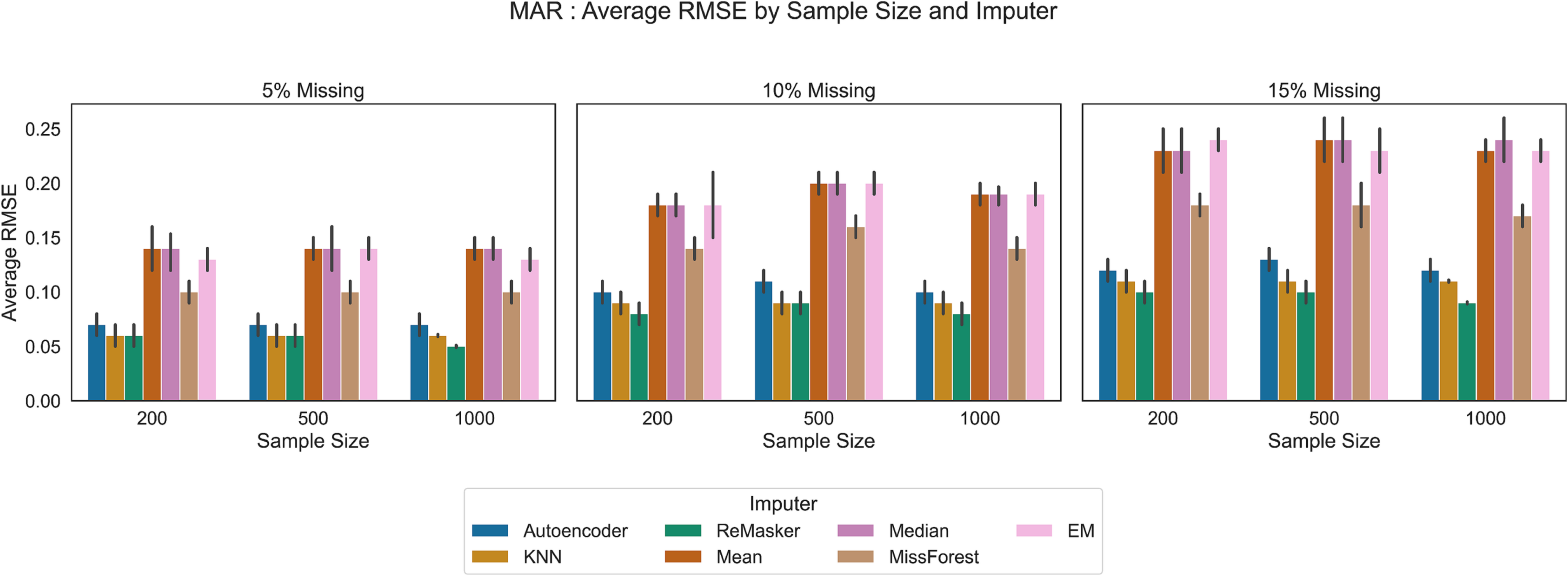
RMSE values in the MAR scenario for all the tested imputation techniques, under varying conditions of sample size and missing ratio.

We compared traditional statistical methods, machine learning techniques, an autoencoder and the ReMasker model across three different sample sizes: 200, 500, and 1,000 observations. Each method was assessed under varying conditions of missingness (5, 10, and 15% missing rates for MAR and MCAR mechanisms). Figures 4, 5 show the results of our analysis.

We started with basic imputation techniques such as mean and median imputation. These methods are advantageous due to their simplicity and ease of implementation. However, they often result in less accurate imputations because they ignore the inherent variability in the data. Also, the EM algorithm shows performances comparable to mean and median imputation. In all the tested scenarios, these traditional methods showed the least favorable performance.

The K-Nearest Neighbor (KNN) and MissForest techniques provided more sophisticated solutions.

MissForest showed comparable error rates to the ReMasker model in MAR scenario and better performance with respect to traditional methods in all scenarios.

KNN imputation, using a distance metric to find similar data points, showed better performance than basic statistical methods and comparable performance to the ReMasker in the MAR scenario with the 5% of missing ratio and the 200 and 500 sample sizes.

The autoencoder approach performed similarly to other machine learning techniques and the ReMasker model. Although its RMSE values were slightly higher than those of these methods, they were still lower than those of conventional imputation methods. For instance, in MAR settings, the autoencoder achieved RMSE values close to the top-performing methods.

The ReMasker model consistently provided the most accurate imputations across all tested scenarios. In particular, it achieves better performances, comparable to MissForest in MCAR scenarios and comparable to KNN in MAR settings.

## 4 Discussion

In this study, we evaluated the efficacy of traditional imputation methods, various machine learning strategies, an autoencoder model, and the ReMasker—a novel Transformer-based model specifically designed for missing data imputation—using a psychometric dataset. Our results show that ReMasker outperforms conventional imputation techniques and exhibits better or comparable performance to other machine learning imputation methods across different scenarios and sample sizes. This performance can be attributed to the Transformer architecture used in ReMasker, which enhances its effectiveness for tabular data imputation. The multi-head self-attention mechanism in Transformer models performs optimizable smoothing over the latent representations of different tokens, making the Transformer robust against severe occlusions or perturbations. This capability is beneficial for learning representations invariant to missing values, thereby improving the imputation process. Indeed, the robust performance of ReMasker across various scenarios suggests it can be used without the need to specifically hypothesize the missing mechanism. In particular, Transformer models excel in such environments because they can capture intricate, nonlinear patterns, thereby preserving essential data structures and minimizing biases in imputation. This adaptability offers a significant advantage for psychometric research, where accurately representing latent constructs is crucial. This flexibility not only improves the precision of imputations but also enhances the reliability of subsequent analyses, leading to more robust insights into psychological constructs and behaviors.

The benefits of ReMasker and similar machine learning models are particularly notable in psychometric contexts, where data is often multidimensional and nuanced, including constructs that involve complex inter-variable relationships. Indeed, the unique value of ML models lies in their adaptability and scalability. These techniques can accommodate large, multidimensional datasets without the need for predefined statistical assumptions, which is especially relevant in psychological research, where data may come from diverse sources (e.g., surveys, behavioral assessments, sensor data) and exhibit multimodal characteristics.

Despite these promising findings, this study has limitations. One limitation is the focus on continuous data imputation, which may not fully represent psychometric datasets, as they often contain categorical data (e.g., Likert scales). This choice was made to maintain consistency across all methods, as not all are capable of handling categorical imputation. Future research should target the development and testing of methods specifically designed for categorical data.

Moreover, this study did not encompass all conventional and deep learning imputation techniques. Techniques such as Multiple Imputation by Rubin (1978) and refined in subsequent studies, which involve generating multiple datasets with different imputed values and combining results for comprehensive analysis, were excluded. These methods, especially Multivariate Imputation by Chained Equations (MICE), represent a robust approach but require specific model specifications that complicate direct comparison with single imputation methods. Also in the deep learning context, some multiple imputation methods have been recently proposed (e.g., Gondara and Wang, 2018; Lu et al., 2020; Vincent et al., 2008). Future research should explore these multiple imputation techniques in psychometric research, potentially integrating deep learning algorithms to enhance their effectiveness.

Additionally, this study did not address the Missing Not At Random (MNAR) mechanism, a scenario that many imputation techniques are not equipped to handle. Future work will aim to develop and test deep learning strategies tailored to the MNAR assumptions in a psychometric context.

Future research should focus on expanding this preliminary work by exploring how ML-based imputation techniques perform across different types of psychometric data and various research contexts, from exploratory analysis to predictive modeling. Conducting systematic simulations across diverse data types, including high-dimensional datasets and those with varying levels of missingness, would clarify the strengths and limitations of specific ML models in psychological settings. Additionally, investigating how imputation methods affect the interpretation of psychometric models in predictive and explanatory frameworks could reveal the nuanced impact of data completeness on psychological insights.

Furthermore, it has to be noted that there are practical challenges associated with implementing ML and deep learning approaches in psychometric contexts. In particular, machine learning and deep learning techniques can require extensive technical knowledge to implement and may be computationally expensive. This issue could be addressed by developing interfaces that make deep learning algorithms more accessible to non-technical users, as in the work of Collier et al. (2024).

## 5 Conclusion

Collectively, the results of this study underscore the critical role of methodological selection tailored to the unique attributes of the dataset and the specific nature of the missing data.

This study underscores the transformative potential of advanced ML models—especially deep learning techniques like the ReMasker model explored in this study—which are capable of learning complex, nonlinear patterns within data, enabling more accurate and flexible imputation approaches that align better with the complexities of psychological and psychometric research.

In conclusion, our evaluation of imputation methods demonstrates that deep learning techniques, in particular the ReMasker model, could improve the missing data imputation by requiring fewer assumptions on data characteristics and opening the possibility of handling complex and multimodal data. This progress is crucial not only for improving the reliability of statistical imputations in psychometric studies but also for enhancing the integrity and validity of research findings across various scientific fields.

## Data Availability

Publicly available datasets were analyzed in this study. This data can be found at: https://osf.io/2ftma/.
